# The Lipophorin Receptor Gene *GdLpR* Regulates Reproductive Diapause in *Galeruca daurica*

**DOI:** 10.3390/insects17060570

**Published:** 2026-05-30

**Authors:** Ling Li, Zhihan Yao, Haichao Wang, Jinghang Zhang, Yanmin Shan, Yanhua Ji, Baoping Pang, Haibin Han

**Affiliations:** 1 Research Center for Grassland Entomology, Inner Mongolia Agricultural University, Hohhot 010018, China; lling@imau.edu.cn (L.L.); 15334730166@163.com (Z.Y.); wanghc@imau.edu.cn (H.W.); 2 Inner Mongolia Autonomous Region Plant Protection and Quarantine Center, Hohhot 010010, China; zhangjinghang0508@163.com; 3 Inner Mongolia Forestry and Grassland Protection Station, Hohhot 010020, China; shanyanmin_1981@163.com; 4 Inner Mongolia Forestry and Grassland Pest Control and Quarantine Station, Hohhot 010051, China; jyh2110@163.com

**Keywords:** *Galeruca daurica*, lipophorin receptor, reproductive diapause

## Abstract

Lipophorin receptors play a crucial role in the reproductive development of insects. However, their regulatory function in insect reproductive diapause remains poorly understood. In this study, a lipophorin receptor gene (*GdLpR*) was cloned from *Galeruca daurica* using RT-PCR. The open reading frame (ORF) of *GdLpR* is 2589 bp in length, encoding 862 amino acids, and possesses typical structural characteristics of the lipophorin receptor family. RT-qPCR analysis indicated that the expression of *GdLpR* is up-regulated in *G. daurica* adults before diapause, exhibits an expression pattern of initial down-regulation, subsequent up-regulation, and then further down-regulation during diapause, and is up-regulated again after diapause termination. After RNA interference of *GdLpR*, the expression levels of diapause-related genes ecdysone receptor (*EcR*), nuclear hormone receptor (*HR3*), and vitellogenin (*Vg*) were downregulated, while the expression level of fatty acid synthase (*FAS*) and total lipid content were upregulated, leading to premature diapause in adult *G. daurica*. These results indicate that *GdLpR* is involved in the regulation of reproductive diapause in *G. daurica*.

## Simple Summary

Reproductive diapause is a heritable state of developmental arrest that adult insects actively enter in response to periodic adverse environmental conditions, which is characterized by arrested ovarian development and internal lipid accumulation. Lipophorin receptors are considered to play critical roles in the regulation of insect reproductive diapause. In the present study, we verified that lipophorin receptors are involved in the reproductive diapause process of *Galeruca daurica*, a serious pest beetle occurring widely in the grasslands of Inner Mongolia, China.

## 1. Introduction

Lipophorin is a non-hexameric storage protein that containing approximately 10% lipids. Its primary function is to transport lipids within insects for metabolism and storage [1]. Lipophorin mainly enter oocytes through endocytosis mediated by lipophorin receptors (LpR), playing a vital role in ovarian development [2]. Since the first *LpR* was successfully cloned from the migratory locust (*Locusta migratoria*) [3], an increasing number of insect *LpRs* have been cloned, such as *Aedes aegypti* [4], *Galleria mellonella* [5], *Bombyx mori* [6], *Blattella germanica* [7], *Nilaparvata lugens* [8], *Acyrthosiphon pisum* [9], and *Mythimna separata* [10]. It has been confirmed that LpR plays a significant role in insect reproductive development. For instance, in *Drosophila*, LpR takes up lipoproteins from oocytes and supplies them to the yolk, serving as the main source of stored lipids [11]. RNAi-mediated silencing of *NlLpR* in *N. lugens* results in delayed ovarian development and reduced fecundity. During oocyte development, LpR regulates the biosynthesis of vitellogenin (*Vg*) by activating S6 kinase, and LpR is transcriptionally regulated by ecdysone, demonstrating that LpR is essential for *Vg* synthesis in the fat body and ovarian development [8].

*Galeruca daurica* has been rampant and caused severe infestations on the grasslands of Inner Mongolia over the past decade or so, posing a serious threat to the healthy development of grassland animal husbandry and ecological security. This beetle has one generation per year and is an oligophagous insect with obligatory diapause. It undergoes summer diapause as adults and overwinters via egg diapause [12,13]. Previous studies in our laboratory have systematically investigated the molecular regulatory mechanisms of its reproductive diapause, revealing that juvenile hormone (JH) plays a regulatory role in reproductive diapause and elucidating the underlying molecular mechanisms [14,15]. Further studies have confirmed that microRNA let-7-5p and miR-2765 regulate the reproductive diapause of *G. daurica* by targeting *Kr-h1* and *FOXO*, respectively [16,17]. The main characteristics of insect reproductive diapause include arrested ovarian development, massive lipid accumulation, and reduced metabolism [18]. However, there is no report on whether *LpR* is involved in the regulation of insect reproductive diapause. This study cloned the *LpR* gene from *G. daurica* and conducted bioinformatics analysis on the gene. RNAi technology was used to investigate its regulatory role in the reproductive diapause of *G. daurica*, aiming to more comprehensively reveal the molecular mechanism of insect diapause.

## 2. Materials and Methods

### 2.1. Test Insects

The eggs of *G. daurica* were collected from the natural grasslands of Siziwang Banner, Inner Mongolia in April 2023. They were incubated and reared in a climate chamber under the conditions of a temperature of 25 ± 1 °C and a photoperiod of 14L:10D. Both the larvae and adults were fed with potted *Allium mongolicum*. The adults on the day of eclosion were used as the test subjects. Meanwhile, the adults at 1, 3, 7, 10, 15, 25, 40, 60, 80, and 100 days post-eclosion were collected, frozen in liquid nitrogen, and then stored at −80 °C. Three biological replicates were set up for each treatment, and each replicate contained 10 adults with a female-to-male ratio (♀:♂) of 1:1.

### 2.2. Gene Cloning

Total RNA was extracted from *G. daurica* adults on the day of eclosion using the Trizol-based RNAios Plus reagent kit (TaKaRa, Dalian, China) according to the manufacturer’s instructions. Briefly, adult samples were ground into powder with liquid nitrogen, homogenized in 1 mL RNAiso Plus, and incubated at 25 °C for 5 min. After centrifugation at 12,000 rpm for 5 min at 4 °C, the supernatant was collected, mixed with 200 μL chloroform, vortexed, and incubated for 5 min. Following centrifugation at 12,000 rpm for 15 min at 4 °C, the upper supernatant was mixed with an equal volume of isopropanol and incubated for 10 min. The RNA pellet was obtained by centrifugation at 12,000 rpm for 10 min at 4 °C, washed twice with 75% ethanol, air-dried, and dissolved in RNase-free water. RNA quality and concentration were detected by 1% agarose gel electrophoresis and a NanoPhotometer™ P-Class ultraviolet spectrophotometer (Implen, Munich, Germany), respectively.

cDNA was synthesized using PrimeScript^TM^ RT reagent Kit with gDNA Eraser (TaKaRa, Dalian, China) according to the manufacturer’s instruction. Based on the transcriptome database of *G. daurica* (PRJNA471603) assembled in our laboratory, specific primers were designed using Primer 5.0 software to amplify the *GdLpR* gene. The PCR amplification system (25 μL) included 1 μL of cDNA template, 1 μL of each forward and reverse primer, 12.5 μL of PCR Master Mix (Promega, Madison, WI, USA), and 9.5 μL of RNase-free H_2_O. The PCR amplification program was as follows: initial denaturation at 94 °C for 5 min; 35 cycles of denaturation at 94 °C for 30 s, annealing at 60 °C for 30 s, and extension at 72 °C for 1 min; final extension at 72 °C for 10 min. The PCR amplification products were detected by agarose gel electrophoresis, then recovered and purified using the MiniBEST Agarose Gel DNA Extraction Kit (TaKaRa, Dalian, China). Subsequently, the purified products were ligated with the pMD-19T vector (TaKaRa, Dalian, China) and transformed into *E. coli* DH5α competent cells (TIANGEN, Beijing, China). The transformed cells were cultured overnight at 37 °C on LB solid medium. On the next day, white single colonies were picked for PCR, and the PCR products were detected by 1.5% agarose gel electrophoresis. Finally, the bacterial liquid samples of positive clones were sent to Beijing Liuhe Huada Gene Technology Co., Ltd. (Beijing, China) for sequencing.

### 2.3. Bioinformatics Analysis

The open reading frame (ORF) of *GdLpR* was identified using the NCBI ORF Finder (https://www.ncbi.nlm.nih.gov/orffinder/) accessed on 18 March 2023. Sequence homology analysis was performed using NCBI BlastP (https://blast.ncbi.nlm.nih.gov/Blast.cgi) accessed on 18 March 2023. The domain organization of the lipophorin receptor was predicted by the SMART algorithm (http://smart.embl-heidelberg.de/) accessed on 31 March 2026. Conservation analysis of the GdLpR amino acid sequence was performed using WebLogo3 (https://weblogo.berkeley.edu/create.cgi) accessed on 20 May 2026, including 9 other Coleoptera insect species (DsLpR: *Diorhabda sublineata*; DvLpR: *Diabrotica virgifera*; LdLpR: *Leptinotarsa decemlineata*; AgLpR: *Anoplophora glabripennis*; TmLpR: *Tribolium madens*; TcLpR: *Tribolium castaneum*; AtLpR: *Aethina tumida*; PpLpR: *Photinus pyralis*; OtLpR: *Onthophagus taurus)*. Phylogenetic reconstruction was performed using the maximum likelihood (ML) method in MEGA 6 [19]. Full-length sequences were adopted for sequence alignment and subsequent phylogenetic analyses. The Jones-Taylor-Thornton (JTT) substitution model was selected, assuming uniform rates among sites. Gaps and missing data were treated with complete deletion. The ML heuristic search used the Nearest-Neighbor-Interchange (NNI) algorithm. Node support was assessed by 1000 bootstrap replicates.

### 2.4. Real-Time Quantitative PCR (RT-qPCR)

RNA extraction and cDNA synthesis were performed as described in Section 2.2. Gene-specific primers were designed using the online software Primer 3 Input (https://www.primer3plus.com/) accessed on 13 April 2023. The succinate dehydrogenase complex (*SDHA*) gene of *G. daurica* was used as the reference gene [20]. The information of primers is shown in Table S1. RT-qPCR reaction system (20 μL): 2 μL of cDNA template, 0.4 μL of each forward and reverse primers, 10 μL of qPCR Master Mix (Promega, Madison, WI, USA), and 7.2 μL of Nuclease-free H_2_O. Reactions were conducted on an FTC-3000P Real-time Quantitative Thermal Cycler (Funglyn Biotech, Canada). Amplification Program: 95 °C for 10 min; followed by 40 cycles of 95 °C for 15 s and 60 °C for 1 min. Melting Curve: 95 °C for 15 s, 60 °C for 15 s, and 95 °C for 15 s. The relative expression levels were calculated using the 2^−ΔΔCt^ method [21]. Three biological replicates and four technical replicates were set up for each treatment.

### 2.5. RNA Interference Experiments

Based on the cloned *GdLpR* sequence of *G. daurica*, the double-stranded RNA (dsRNA) synthesis primers were designed using Primer 5.0 software. The T7 promoter sequence (5′-TAATACGACTCACTATAGGG-3′) was added to the 5′-end for PCR amplification. PCR amplification was performed using cDNA from female adults as the template. The PCR product was ligated into the pGEM^®^-T Easy vector (Promega, Madison, WI, USA), followed by transformation and screening of positive clones, and then sequencing was performed (method as described in Section 2.2.). The dsRNA of *GdLpR* (dsGdLpR) was synthesized using the T7 RiboMAX™ Express RNAi System Kit (Promega, Madison, WI, USA). Its quality was determined using 1% agarose gel electrophoresis and a NanoPhotometer™ P-Class ultraviolet spectrophotometer (Implen, München, Germany). Finally, dsGdLpR was diluted to 1000 ng/μL and stored at −80 °C for later use. dsGFP was synthesized following the same procedure as the target gene. The lengths of synthesized dsGdLpR and dsGFP were 445 bp and 419 bp, respectively. The above fragment lengths exclude the T7 promoter sequences, referring only to the specific targeted gene regions.

2 μL of dsGdLpR was injected into the body of female adults of *G. daurica* on the first day after eclosion, via the intersegmental membrane between the 2nd and 3rd abdominal segments, using a microinjection instrument (SHIMADZU, Kyoto, Japan). dsGFP was synthesized and injected as the negative control, and non-injected individuals served as the blank control. At 24, 48, 72, and 96 h after dsGdLpR injection, the RNAi efficiency was detected by RT-qPCR to determining the optimal time point for maximum RNAi efficiency. RT-qPCR was also used to detect the effects of *GdLpR* silencing via RNAi on the expression levels of diapause-related genes, including ecdysone receptor (*EcR*), fatty acid synthase (*FAS*), and *Vg*. Each treatment had three biological replicates, with each replicate consisting of six female adults.

### 2.6. Determination of Lipid Content and Observation of Development

Lipid content was measured at 24, 48, 72, and 96 h after injection of dsGdLpR and dsGFP, respectively. The chloroform-methanol method was used to measure the total lipid content [22]. Each treatment included three biological replicates, with 20 female adults per biological replicate, and measurements were performed on individual insects. The detailed procedures were as follows: (1) Adult *G. daurica* were rinsed with deionized water, dried with filter paper to remove surface moisture, and weighed for fresh mass (FM); (2) Samples were dried in an oven at 65 °C for 72 h to a constant weight, and dry mass (DM) was recorded; (3) The insect bodies were ground into powder, homogenized in 1 mL chloroform-methanol mixture (V:V = 2:1), centrifuged at 2600 rpm for 10 min, and the supernatant was discarded; (4) Step 3 was repeated; (5) The remaining residue was dried in an oven at 60 °C for 48 h to a constant weight, and lean dry mass (LDM) was measured.

Lipid content = (DM − LDM)/LDM.

Previous studies in our laboratory have shown that adult *G. daurica* cease feeding and enter diapause approximately one week after eclosion, during which their respiratory intensity decreases sharply [12]. Therefore, in this experiment, the feeding behavior of adult *G. daurica* was observed twice daily after injection, and the number of feeding adults was recorded each day. Adults were considered to be in diapause when they began to stop feeding continuously. Each treatment consisted of three biological replicates, with 50 female adults per replicate.

### 2.7. Data Analysis

Significance analysis of differences was performed using Duncan’s test or *t*-test in one-way analysis of variance (ANOVA) with SPSS 20.0 software (* *p* < 0.05, ** *p* < 0.01).

## 3. Results

### 3.1. Cloning and Sequence Analysis of GdLpR from G. daurica

**Figure 1:**
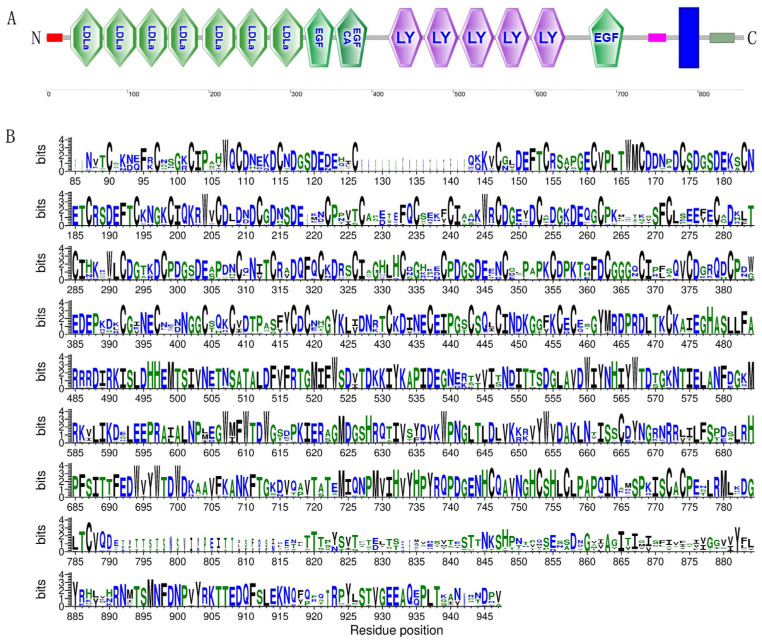
Analysis of the amino acid sequence of GdLpR in *G. daurica*. (**A**) Domain organization of GdLpR in *G. daurica*. Each symbol indicates a specific motif, including signal sequences (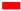), Low-density lipoprotein receptor domain class A (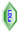), Epidermal growth factor-like domain (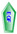). Low-density lipoprotein-receptor YWTD domain (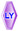), low compositional complexity (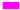), transmembrane helix region (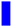). (**B**) Conserved motifs of LpR from *G. daurica* and 9 other Coleoptera insects.

**Figure 2:**
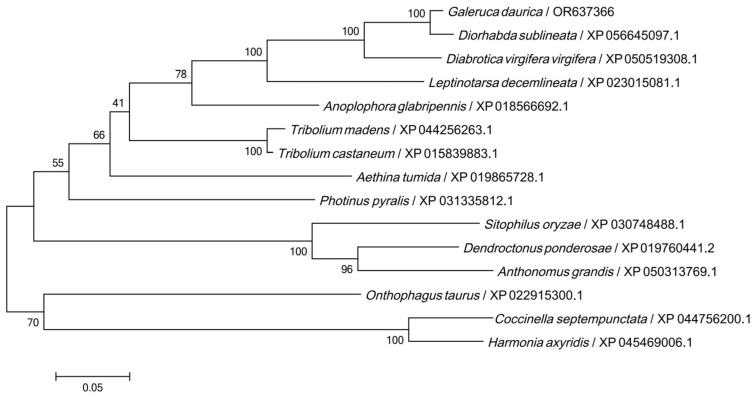
Phylogenetic tree of LpR from *G. daurica* and other insects based on amino acid sequences.

The full-length ORF sequence of the *LpR* gene from *G. daurica* was cloned via RT-PCR and named *GdLpR* (GenBank accession number: OR637366). The ORF is 2589 bp in length and encodes 862 amino acids. SMART analysis revealed that it belongs to the low-density lipophorin receptor family and possesses the typical structural characteristics of this family (Figure 1A), including seven low-density lipophorin receptor (LDLR) domains, two epidermal growth factor precursor homology domains, one calcium-binding epidermal growth factor domain, and five YWTD repeat domains. In addition, it contains one transmembrane domain and one cytoplasmic domain at the C-terminus. The amino acid sequence of LpR is highly conserved (Figure 1B). Amino acid sequence alignment showed that LpR from *G. daurica* shared the highest identity (96.99%) with that LpR from *Diorhabda sublineata*, a species from the same family; followed by the identity with LpR from *Diabrotica virgifera*, which was 86.52%. In addition, the amino acid identities with the LpRs of *Leptinotarsa decemlineata*, *Anoplophora glabripennis*, *Tribolium madens*, *Onthophagus taurus*, *Tribolium castaneum*, *Aethina tumida*, and *Photinus pyralis* were 77.43%, 72.02%, 71.94%, 67.40%, 66.77%, 66.56%, and 65.62%, respectively (Figure S1). Phylogenetic analysis indicated that GdLpR had the closest genetic relationship with DsLpR from *D. sublineata*, with a bootstrap support of 100% (Figure 2).

### 3.2. Expression Analysis of GdLpR at Different Developmental Stages of G. daurica Adults

**Figure 3:**
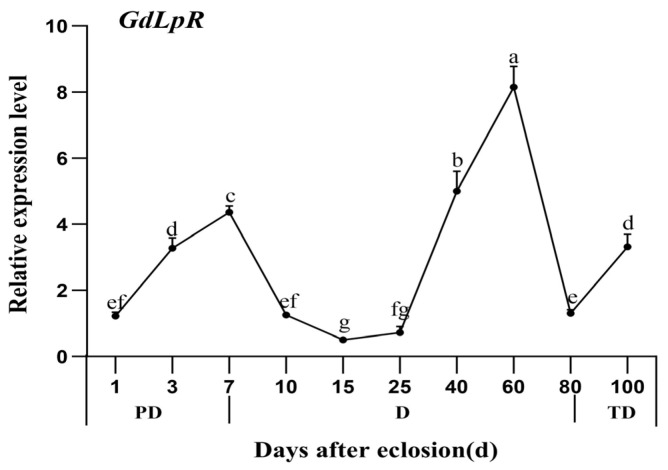
Expression pattern of *GdLpR* in different developmental stage of adult *G. daurica.* PD: Before diapause; D: Diapause period; TD: After diapause. Duncan’s multiple range test was used for differential analysis, and different letters indicate significant differences at *p* < 0.05.

As shown in Figure 3, *GdLpR* was expressed at all adult stages. The expression level of *GdLpR* showed an upward trend before diapause (1–7 days post-eclosion); during diapause, it first decreased (7–15 days post-eclosion), then increased (15–60 days post-eclosion), and decreased again (60–80 days post-eclosion); after diapause termination (after 80 days post-eclosion), the expression level was up-regulated once more.

### 3.3. Silencing Efficiency of GdLpR Gene by RNAi

**Figure 4:**
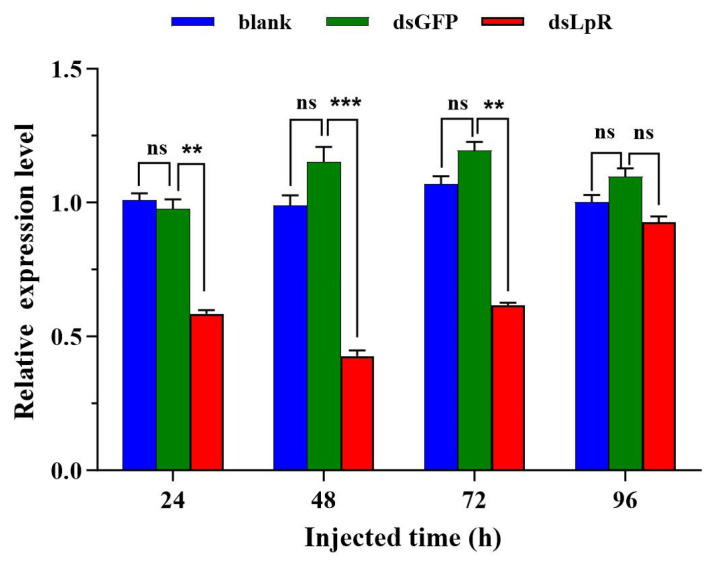
Silencing efficiency of *GdLpR* by RNAi in *G. daurica.* Differential analysis was performed using the *t*-test. **: *p* < 0.01; ***: *p* < 0.001; ns: no significant difference.

To detect the silencing efficiency of injected dsRNA on the *GdLpR* gene, RT-qPCR was used to determine the gene expression levels at 24, 48, 72, and 96 h post-injection. The results showed that there was no significant difference between the negative control group injected with dsGFP and the blank control group without injection, indicating that the negative control was reliable. Compared with the negative control group (dsGFP), the silencing efficiency of dsLpR at 24, 48, 72, and 96 h post-injection were 35.96%, 83.24%, 41.23%, and 11.82%, respectively. This indicates that the silencing efficiency is optimal at 48 h after interference (Figure 4).

### 3.4. Effects of GdLpR Silencing on Reproductive Diapause in G. daurica

#### 3.4.1. Effects of *GdLpR* Silencing on Diapause-Related Genes

**Figure 5:**
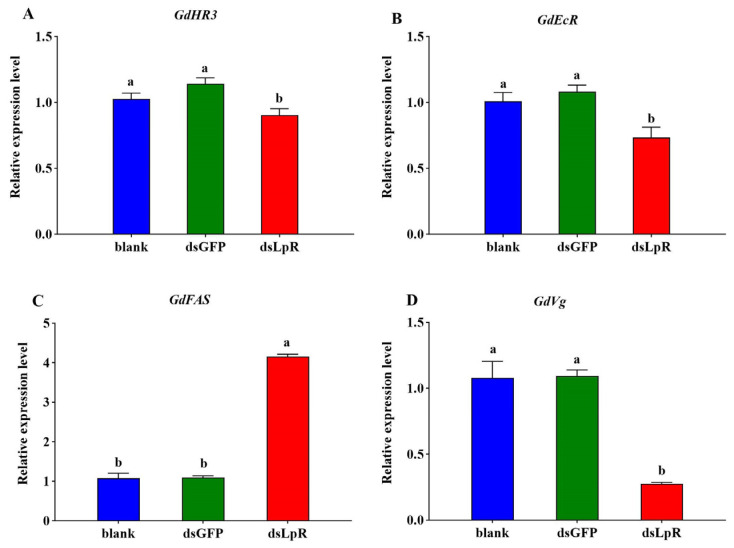
Effects of silencing *GdLpR* on diapause-related gene expression. (**A**) The transcriptional levels of *GdHR3*; (**B**) The transcriptional levels of *GdEcR*; (**C**) The transcriptional levels of *GdFAS*; (**D**) The transcriptional levels of *GdVg.* Differential analysis was performed using Duncan’s test, *p* < 0.05; different letters indicate significant differences.

At the optimal silencing efficiency time (48 h), the effects of *GdLpR* silencing on diapause-related genes were detected (Figure 5). The results showed that compared with the negative control (dsGFP), the transcriptional levels of *GdHR3* (Figure 5A), *GdEcR* (Figure 5B), and *GdVg* (Figure 5D) were significantly down-regulated (*p* < 0.05), with their relative expression levels decreased by 20.83%, 32.2%, and 74.73%, respectively; while the transcriptional level of *GdFAS* (Figure 5C) was up-regulated by 2.8 folds (*p* < 0.05).

#### 3.4.2. Effects of *GdLpR* Silencing on Total Lipid Content

**Figure 6:**
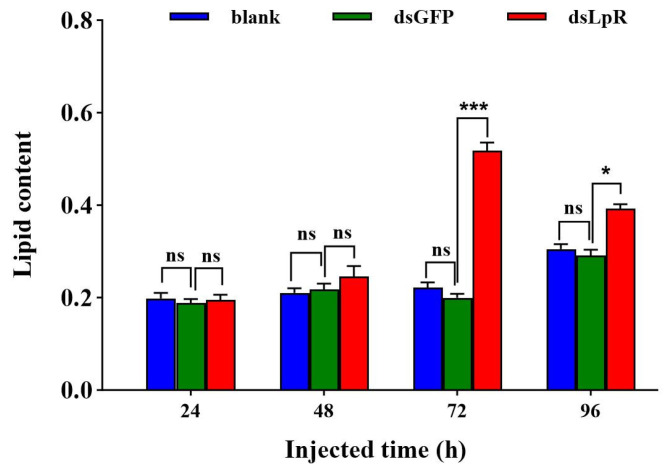
Effect of silencing *GdLpR* on total lipid content in *G. daurica*. Differential analysis was performed using the *t*-test. *: *p* < 0.05; ***: *p* < 0.001; ns: no significant difference.

As shown in Figure 6, compared with the control groups, the total lipid content of the test insects showed no significant change at 24 h and 48 h after injection of dsGdLpR; it increased extremely significantly at 72 h after injection (*p* < 0.01); and the total lipid content also increased significantly at 96 h after silencing *GdLpR* (*p* < 0.05).

#### 3.4.3. Effects of *GdLpR* Silencing on Diapause Onset

**Figure 7:**
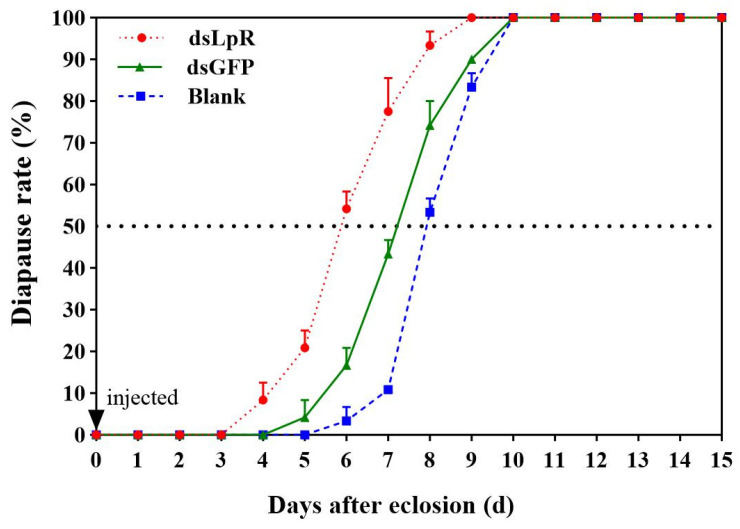
Effect of silencing *GdLpR* on reproductive diapause in *G. daurica*.

As shown in Figure 7, there were differences in the diapause initiation time of *G. daurica* adult among different treatment groups. Adults injected with dsLpR began to enter diapause on the 3rd day; adults injected with dsGFP initiated diapause on the 4th day; while adults in the non-injected group did not start diapause until the 5th day. As time elapsed after injection, the cumulative diapause rate of each group increased gradually, and the diapause process of the dsLpR treatment group was the fastest. Specifically, the time for the diapause rate of the dsLpR group to reach 50% was 5.89 days, which was 1.33 days and 2.1 days earlier than that of the dsGFP group (7.22 days) and the blank control group (7.99 days), respectively.

## 4. Discussion

LpR belongs to the low-density lipophorin receptor superfamily and is one of the transporters responsible for delivering nutrients required for embryonic cell development during insect reproduction [2]. In this study, an *LpR* gene was cloned from *G. daurica* for the first time and named *GdLpR*. Sequence analysis revealed that it possesses the conserved structural characteristics of this family, namely, LDLR domain, epidermal growth factor (EGF) precursor homology domain, EGF calcium-binding domain, YWTD repeat domain, O-glycosylation domain, transmembrane domain, and cytoplasmic domain [1].

The main characteristics of insect reproductive diapause in insects are arrested ovarian development and massive lipid accumulation [18]. In *Apis mellifera*, the putative lipophorin receptor being downregulated as the ovaries are activated [23]. Vg is the most abundant yolk protein in insect eggs, and its synthesis, secretion, and incorporation into developing oocytes play crucial roles in the reproductive development of female insects [24,25]. In the majority of insects, precursor vitellogenin (Vg) and lipophorin (Lp) are synthesized extraovarially in the fat body and subsequently internalized by competent oocytes via membrane-bound receptors (i.e., vitellogenin receptors (VgRs) and lipophorin receptors (LpRs), respectively) [2]. Lu et al. [8] found that *LpR* is essential for Vg synthesis in the brown planthopper (*N. lugens*), and RNAi-mediated silencing of *NlLpR* effectively reduced Vg synthesis, thereby decreasing the fecundity of *N. lugens*. In this study, silencing *GdLpR* down-regulated the expression of *GdVg*, suppressed the reproductive development of *G. daurica*, and consequently induced reproductive diapause.

Lipids are fundamental for various life activities such as insect growth and development, and lipid synthesis and metabolism influence insect diapause. Before entering diapause, insects need to accumulate abundant energy reserves including lipids, which provide nutrients and energy sources during diapause [26]. In *Locusta migratoria*, the neutral lipid content was significantly decreased in the ovary after RNAi against *LmLpR*, which led to a retarded ovarian development [27]. FAS is a key enzyme in lipid biosynthesis that converts acetyl-CoA into palmitate, thereby facilitating the synthesis of triglycerides [28]. In the present study, silencing *GdLpR* in adult *G. daurica* prior to diapause upregulated the expression of *GdFAS*, promoted lipid accumulation, induced reproductive diapause, and inhibited reproductive development.

Juvenile hormone (JH) is one of the most important hormones regulating insect growth, development, reproduction, and diapause [29]. Previous studies in our laboratory have shown that injection of the JH analog methoprene into *G. daurica* adults before diapause up-regulated the expression of *Vg*, promoted ovarian development, down-regulated the expression of *FAS*, and inhibited lipid accumulation, thereby inducing reproductive development and suppressing reproductive diapause [14]. In contrast, RNAi-mediated silencing of the JH signaling pathway genes *Met* and *Kr-h1* yielded opposite results [15,16]. Liu et al. [30] obtained similar results in their study on the reproductive diapause of *Colaphellus bowringi*. It has been reported that JH up-regulates the protein level of *LpR* in the fat body of the German cockroach [7]. However, whether JH can regulate reproductive diapause in adult *G. daurica* by upregulating the expression of *LpR* remains to be further studied.

20-hydroxyecdysone (20E) plays diverse regulatory roles during insect reproduction [31]. In *Drosophila melanogaster*, 20E regulates lipid accumulation and oocyte development through *EcR* [32,33]. Supplementation of 20E in the diet greatly activated LpR protein synthesis in *G. mellonella* [5]. In *N. lugens*, RNAi-mediated knockdown of *EcR* significantly down-regulated the expression of *LpR*, whereas 20E application upregulated *LpR* expression [8]. In the present study, silencing *LpR* significantly reduced the expression of the 20E signaling pathway genes *EcR* and *HR3*. This expression change is not derived from direct upstream regulatory relationship, but is probably mediated by indirect metabolic feedback effects in physiological processes, which is commonly observed in the hierarchical 20E signaling pathway [34]. Previous studies in our laboratory demonstrated that 20E application before reproductive diapause significantly upregulated the expression of *EcR*, *HR3*, and *Vg*, but downregulated *FAS* expression and lipid accumulation in adult *G.daurica*, thereby stimulating reproductive development and suppressing reproductive diapause [35]. However, whether 20E can regulate reproductive diapause in *G. daurica* via *GdLpR* remains to be further investigated. In subsequent studies, we will further expand the experimental design, conduct rescue experiments of exogenous 20E and JH agonists on the *GdLpR* knockdown phenotype, and deeply explore the molecular mechanism by which *LpR* regulates reproductive diapause under the regulation of hormone signals.

## 5. Conclusions

This study first cloned the *GdLpR* gene from *G. daurica*, which has conserved structural characteristics of the LDLR superfamily. Silencing *GdLpR* downregulates *GdVg* expression, upregulates *GdFAS* expression, promotes lipid accumulation, inhibits reproductive development, and thereby induces reproductive diapause in *G. daurica* adults. Additionally, *GdLpR* silencing significantly reduces the expression of 20E signaling pathway genes *EcR* and *HR3*. Although previous studies have shown that JH and 20E regulate insect reproductive diapause and are associated with *LpR* in other insect species, whether JH and 20E regulate reproductive diapause in *G. daurica* through *GdLpR* requires further investigation.

## Data Availability

The original contributions presented in the study are included in the article/Supplementary Materials; further inquiries can be directed to the corresponding author(s).

## Supplementary Materials

The following supporting information can be downloaded at: https://www.mdpi.com/article/10.3390/insects17060570/s1, Figure S1: Amino acid sequence alignment of LpR from *G. daurica* and 9 other Coleoptera insects; Table S1: Primer information.

## Abbreviations

The following abbreviations are used in this manuscript:

LpR	lipophorin receptor
EcR	ecdysone receptor
HR3	nuclear hormone receptor
Vg	vitellogenin
FAS	fatty acid synthase
LDLR	low-density lipophorin receptor
EGF	epidermal growth factor
YWTD	Tyr-Trp-Thr-Asp motif
