# Supplementary material for: The Lipophorin Receptor Gene GdLpR Regulates Reproductive Diapause in Galeruca daurica
Source: Insects. 2026 May 30;17(6):570. doi: 10.3390/insects17060570 (PMC13299740; doi:10.3390/insects17060570)
Supplement: Supplementary file 1 [file insects-17-00570-s001.zip › Table S1.pdf]

Table S1. Primer information.

**Primer Name**	**Primer sequence（5'-3'）**	**Purpose**
LpR-F	TTTACCCCTCCTCTTGAGGGTATCC	Cloning
LpR-R	GCTCTTTTGGTTATGTACTGGCTCG	
qLpR-F	ACAACGTCCGATGGTTTAGC	RT-qPCR
qLpR-R	CATTCCGGCACGTTCTATTT	
qEcR-F	CTACACATTCCGGCCTCATT	
qEcR-R	AGGACCAACACCTCGACAAC	
qHR3-F	GAAAGCGTCTTGGGGTAACA	
qHR3-R	CGATTGCGCACAGAAACTTA	
qFAS-F	GAAGCGTTATGGTCCCATGT	
qFAS-R	GAACAAGACTGCCGAGAACC	
qVg-F	TTGGTTGAACAGCAGCTTTG	
qVg-R	GGACACTGTTTTGCCCCTAA	
qSDHA-F	GGGAGACCACAATCTCCTCA	
qSDHA-R	AGGTGGTGGTCCTAAGTCCA	
ds*LpR-F*	TAATACGACTCACTATAGGGGCGCACCGAAGACTTTTTAG	RNAi
ds*LpR-R*	TAATACGACTCACTATAGGGCCGTCCAGTACACCCAGTCT	
ds*GFP-F*	TAATACGACTCACTATAGGGCACAAGTTCAGCGTGTCCG	
ds*GFP-R*	TAATACGACTCACTATAGGGTTCACCTTGATGCCGTTC	

The underlined sequence represents the T7 promoter.
