# Supplementary figures and images for: The Lipophorin Receptor Gene GdLpR Regulates Reproductive Diapause in Galeruca daurica
Source: Insects. 2026 May 30;17(6):570. doi: 10.3390/insects17060570 (PMC13299740; doi:10.3390/insects17060570)

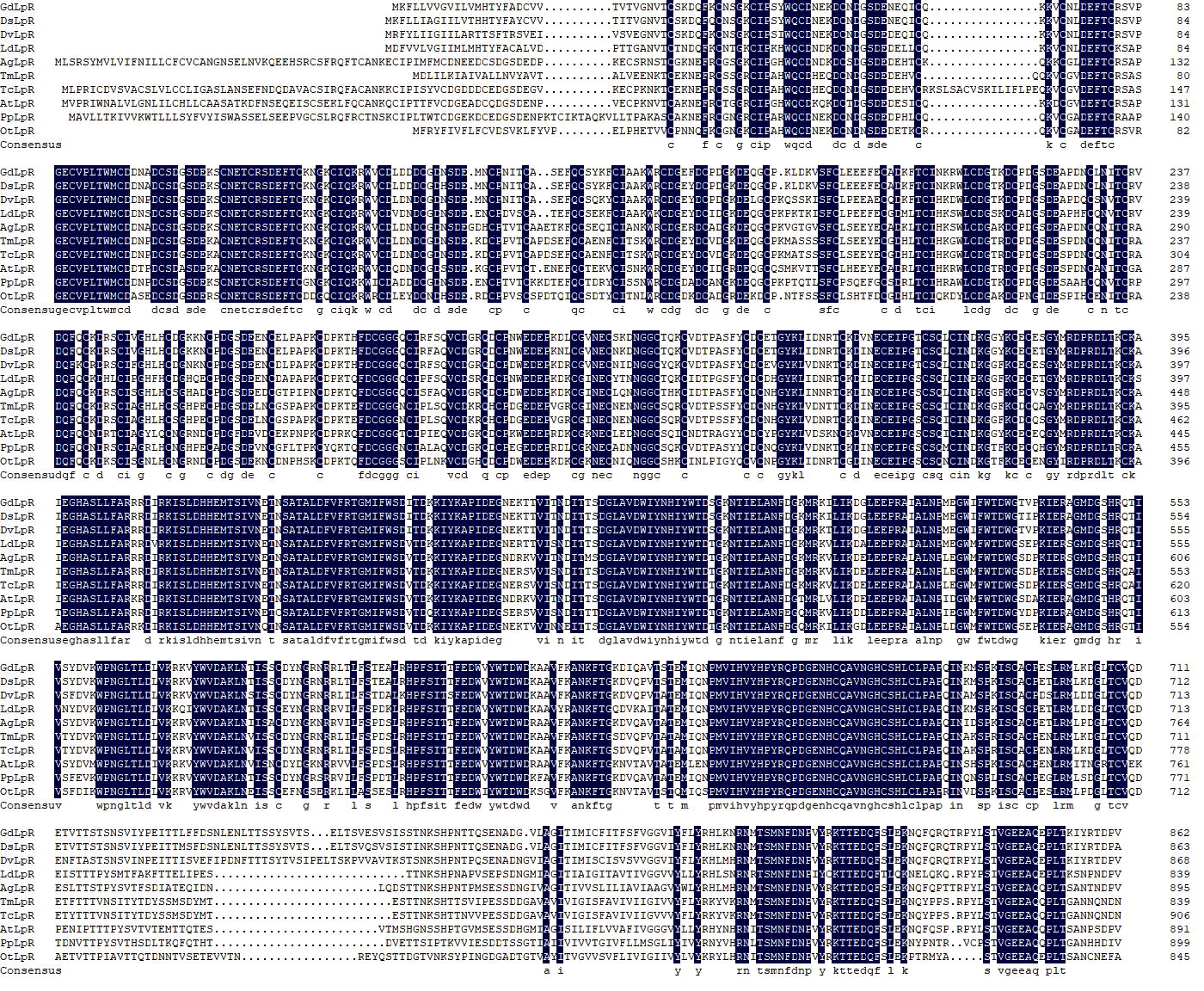

Supplement: Supplementary file 1 [file insects-17-00570-s001.zip › Figure S1.jpg]
